# Evaluation of pharmaceutical pictogram comprehension among adults in the Philippines

**DOI:** 10.1186/s40545-022-00426-y

**Published:** 2022-04-07

**Authors:** Margarita M. Gutierrez, Chanthawat Patikorn, Puree Anantachoti

**Affiliations:** 1 Present Address: Department of Pharmacy, University of the Philippines Manila College of Pharmacy, Manila, Philippines; 2 Department of Social and Administrative Pharmacy, Faculty of Pharmaceutical Sciences, Chulalongkorn University, Bangkok, Thailand

**Keywords:** Comprehensibility, Pharmaceutical pictograms, Medication literacy, Pictograms, Visual aids, Medication information, Philippines, Health literacy, Pharmacy, Patient education

## Abstract

**Background:**

The use of pharmaceutical pictograms to enhance patients’ understanding of drug regimens has been proven effective in many countries. There are two reference systems for pictograms generally used in pharmacy: the United States Pharmacopeia (USP) and International Pharmacy Federation (FIP). This study aimed to evaluate the effectiveness of USP and FIP pictograms among adults in the Philippines by identifying how many pictograms would pass the American National Standards Institute (ANSI) criterion of 85% comprehension, and to describe the factors affecting pictograms’ comprehension.

**Methods:**

A descriptive cross-sectional research using a face-to-face interview was performed to evaluate 108 pictograms in 52 Filipino adults enrolled through quota sampling. Descriptive statistics, Mann–Whitney *U* test (Wilcoxon rank-sum test), univariate linear regression, and multiple linear regression were used to statistically analyze the data collected.

**Results:**

Only 17 (16 USP and 1 FIP) out of the 108 pictograms (15.74%) passed the ANSI criterion. The median score of Filipinos was 71 out of 108 pictograms (Interquartile range: 10–96). The multivariate model (*R*^2^ = 0.5645, *F* (4,47) = 15.23) suggested that the score was lower by 5.85 points if the user was female, 21.58 points lower if the participant was below Grade 12 education level, and 1.20 points lower if the patient was greater than 46 years old. Education level was identified as the significant predictor (*p*-value < 0.0000*, power = 99.98%). The participant with greater than Grade 12 has a higher comprehension score of rank-sum 952.5 (Expected = 689) compared to only 425.5 (Expected = 689).

**Conclusions:**

Since only 17 pictograms passed as stand-alone tool for patient information material, the researchers recommend the use of verbal and written instructions to complement pictograms to enhance comprehension. Furthermore, the government should consider the inclusion of health pictograms in basic health education.

**Supplementary Information:**

The online version contains supplementary material available at 10.1186/s40545-022-00426-y.

## Background

Pictograms, which come from the Latin word *pictus* that means “painted” and the suffix––graph that means “something written,” are pictures that represent a word or phrase [1, 2]. They are a form of communication that provides meaning through its pictorial resemblance to a physical object or an action that is, in theory, easier to use other than reading written instruction [3, 4]. Lack of compliance with the prescribed treatment plan due to communication barriers between healthcare professionals and patients is one of the contributing factors for treatment failure [4]. One of the interventions used in many countries to improve this is the use of pictograms in patient information materials. Pictograms are used and previously researched, both alone and in combination with text, on the acquisition, comprehension, and recall of information [5]. Pictograms in previous researches had shown a positive effect on the acquisition and comprehension of drug information, decreased medication dosing errors, and improved adherence [4, 6]. For example, pictograms have a proven value when used in a pill card (medication instruction) for the improvement of adherence to antihypertensive medications in patients with hypertension [7]. In previous researches, simple pictorials on medication information supported by verbal instructions were better comprehended by individuals with low literacy skills and the elderly [8, 9].

There are two reference systems for pictograms generally used in pharmacy: the United States Pharmacopeia (USP)[10] and International Pharmacy Federation (FIP) [11]. USP offers 82 pictograms that are downloadable for free after accepting the license agreement [10]. The USP pictograms are standardized graphic images designed to help convey medication instructions, precautions, and/or warnings to patients and consumers and have been widely used in Western countries [10]. However, published studies regarding their usability and legibility in different settings like in South Africa revealed potential limitations [4]. In contrast, FIP pictograms developed in June 2009 were pre-tested in a diverse population [11]. They were last updated in February 2017 to fix issues with comprehension [4]. In the current study, pictograms from both sources were evaluated according to the international standards for comprehensibility of pictorial symbols to assess the suitability for Filipinos.

In the Philippines, household remedies, over-the-counter drugs, and herbal medicines, and traditionally used herbal products are required by the Food and Drug Administration (FDA) to submit a Patient Information Leaflet (PIL) for registration. On the other hand, all prescription medicines, new chemical entities (NCEs), biological products, and herbal medicines are required to submit a Package Insert (PI). The difference between the two is that PI is intended for use by healthcare professionals, while PIL is intended for use by patients and is written in layman's language [12]. The use of pictograms for both the PIL and PI is not a requirement. Only specific household and urban hazardous substances are required by the law to include pictograms [13]. The system used is the 2003 Globally Harmonized System (GHS) of Classification and Labeling of Chemicals which was adopted in the Philippines in 2009 under Administrative Order No. 01 series of 2009 [13]. Thus, the use of USP and FIP pictograms may be uncommon to Filipinos and this in turn, may affect their ability to comprehend the pictograms when used in patient information materials.

Pictogram comprehension can be varied significantly in different countries [14]. Testing of pharmaceutical pictograms is necessary because of differences in cultures and demographics which may cause people to understand the meaning of images differently. This is consistent with the recommendation of Kassam (2004), where they stated that “Taking into account the culture of the target population is essential.”[15] Furthermore, it was recommended by van Beusekom, Kerkhoven, Bos (16) that it is essential to involve the intended target group in the evaluation of pictograms and pictogram-enhanced information because it was seen that different audiences can vary considerably in how they perceived and responded to pictograms [16, 17].

Currently, there is no published literature conducted in the Philippines to assess the effectiveness of pictograms. In international researches, however, they have found that a variety of factors can impede effective communication, compromising patient safety when using pharmaceutical products. Poverty, literacy, age, and patient preferences are only a few of the factors to consider [18]. Pictograms have been shown in many countries to be successful in improving patients' understanding of drug regimens, which could help close this gap [19].

Although pharmaceutical pictograms from both USP and FIP are available for use, there was no study conducted to test pictogram comprehension among Filipinos. This study aims to evaluate pharmaceutical pictograms, specifically the USP and FIP, among adults in the Philippines. This would validate if these materials are a feasible form of pharmaceutical communication that may be used for patient information materials in the Philippines. Specifically, the research objectives of the study are 1) to determine which of the 108 pictograms from USP and FIP would pass the ANSI standard of 85% criterion for comprehension when tested in Filipino adults and 2) to describe the factors that associated with pictogram comprehension of Filipinos.

## Main text

### Materials and methods

#### Study design

The research design was a cross-sectional study. The data collection procedure was a face-to-face interview of Filipino adults residing in the National Capital Region of the Philippines between the data collection period of January to February 2021. The study protocol was approved by the University of the Philippines Manila Research Ethics Board (UPMREB) Review Panel [UPMREB CODE: 2020-745-01]. The participants were informed about the study after which written informed consent was obtained from participants who agreed to participate.

#### Population sampling

Participants must be at least 18 years old, Filipino citizens, and must not be studying or working in any healthcare professional field. The sample size was computed using the formula $n = \frac{{Z_{{\alpha/2}}^{2}\,p(1 - p)}}{{e^{2} }},$ where *α* = 0.05, *Z*_α⁄2_ = 1.96, *p* = 0.85 (based on the ANSI criterion, proportion of citizens who understand the meaning of symbolic images correctly), and margin of error = 0.1. While the result of the computation was 49, the researchers opted to increase the sample size to 52. A stratified quota sampling was used. Participants were divided into four groups based on age (18–45 years old vs. 46 years and older) and education level (up to Grade 12 and greater than Grade 12).

#### Pictograms evaluation

A total of 108 pharmaceutical pictograms were included in this study, including all 82 pictograms developed and disseminated by the USP Dispensing Information [10] and 26 pictograms from the FIP [11]. To minimize the burden to the participants, only FIP’s pictograms representing the same meaning as the USP’s pictograms were selected. Moreover, only one pictogram among pictograms with similar meaning was selected, such as pictogram for “take 2 times a day” was selected to represent pictogram for “take 3 times a day” and “take 4 times a day.”

**Fig. 1:**
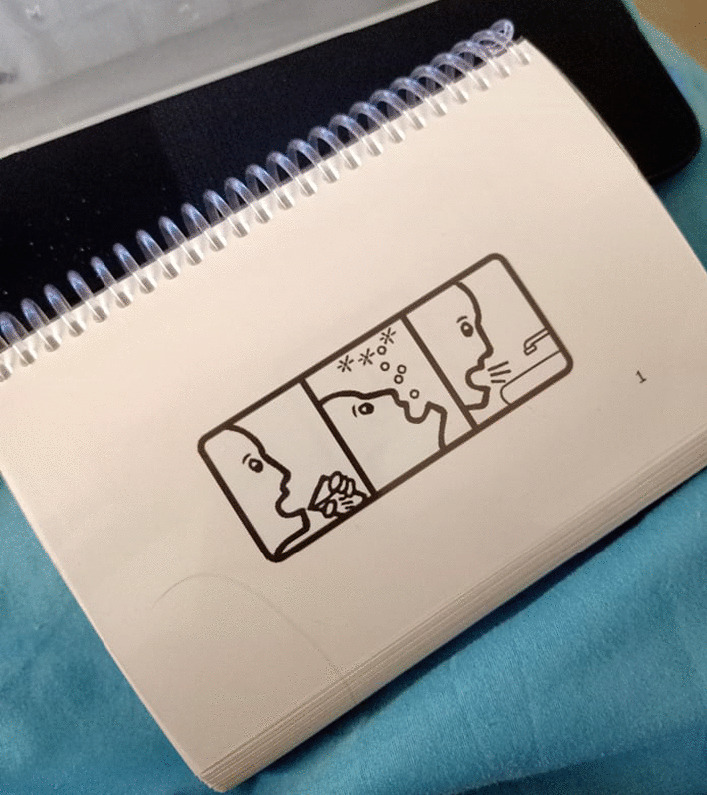
Pictogram flipbook

For data collection purposes, the pictograms were printed in random order. The height of the pictograms was 1 inch in a 108-page flipbook (1 pictogram per page) as shown in Fig. 1. The size of the pictograms was designed to ensure that visual acuity would not be a factor. The order of appearance of the pictograms in the flipbook was the basis for a pictogram ID number (the first pictogram has an ID number of #1).

#### Data collection

The researchers invited participants from the national capital region of the Philippines by face-to-face interview, they were asked if they were willing to participate in a 30–45 min survey. All participants who agreed were screened based on the inclusion and exclusion criteria after collecting the information, such as age, gender, education level, and field of practice or education. Those who were qualified were given a short orientation related to the research and ethical considerations, after which they were asked to sign a consent form.

After that, the researchers asked the participants to view and interpret the 108 pictograms through the flipbook within the time limit of 20 s per pictogram. All participants answered an open-ended question in which the respondents were asked to interpret the meaning of each pictogram. The response may be in English or Filipino. The answers of the participants were video recorded for the accurate encoding of responses and time took. The pharmacist researchers who are fluent in English and Filipino evaluated the responses in terms of correctness. The time taken for the participants to give a response per pictogram was also calculated. The analyst encoded the data by giving “1” points for all the correct answers and “0” for a wrong answer. If the participants took more than 20 s to respond, such an answer was marked as incorrect or “0.”

#### Data analysis

For the first objective, the researchers used descriptive statistics to report the total score and time spent for each pictogram. Per pictogram, the sum of the score was computed divided by 52. The researchers then computed the % of the population that passed to evaluate whether the pictogram passed the ANSI criterion by multiplying it to 100. The criterion for acceptance was based on the ANSI Z535.326 which determined that an image to communicate effectively should be correctly understood by at least 85% of the population (45 out of 52 participants can interpret the pictogram correctly) [20]. Pictograms that got a comprehension score of 85% or higher were the pictograms that were deemed “Passed”; otherwise, they were considered “Failed.” The pictograms were also categorized based on the quartile score where the failed pictograms but in the upper quartile were identified. The pictograms in the lower quartile were the pictograms that required extra precaution. The researchers also performed a descriptive analysis of pictograms that were misinterpreted (answered by patients within 5 s or 1/4 of the allotted time but were wrong) to evaluate the common errors.

For the second objective, the total scores of the 52 participants were collected. The comprehension scores of participants were computed by adding all the correct responses of the participant, while the average time of participants was computed as the total number of times spent before providing the response divided by 108 pictograms. To determine the factors associated with the total score, the Shapiro–Wilk test for normality, Mann–Whitney U test (Wilcoxon rank-sum test), and univariate linear regression were conducted. Multivariate linear regression of all factors was also conducted to confirm the associated factors in relation to other variables. The dependent variable was the total comprehension score of participants, while the independent variables were sex, education, age, and average time in seconds. The level of statistical significance for independent variables was set at 0.05.

## Results

The 52 participants comprised mostly female (57.69%, *n* = 30) with an average age of 42 years old (SD = 11.90, range 18–67 years old).

### Pictogram comprehension

Only 17 out of the 108 pictograms (15.74%) passed the ANSI 85% criterion, 16 from the USP and 1 from the FIP. The pictogram #56 (USP) “Wash hands” was correctly interpreted by all 52 participants, while none of them could interpret pictogram #6 (USP) “Are you taking any other medicines?”.

### Pictograms that passed

One of the pictograms that passed the ANSI criterion is pictogram #52 “For headache” from FIP with a correct response of 98.08%. Notably, the USP pictograms #11 and #64 also represented “For headache,” but these two pictograms were passed with lower scores compared to the FIP, thus suggesting that it was preferred to use #52 for headache indication than other pictograms. It was also worth noting that “for headache” was the only pictogram for indication purposes that passed ANSI 85% criterion. The pictograms that passed are compiled in Table 1. The passed pictograms were deemed understandable by the majority of Filipinos; therefore, they may be used in PIL or PI as stand-alone material.

**Table 1:** Pictograms that Passed

Pictogram ID # and Image	Meaning	Source	% Patients correctly answer
Administration			
21.	Place drops in the lower eyelid	USP	86.54
53.	Wash hands/Place drops in nose/Wash hands again	USP	88.46
105.	For injection	USP	86.54
Regimen			
20.	Take 2 times a day	USP	92.31
50.	Take at bedtime	USP	88.46
90. InlineMediaObject>	Take with meal	USP	92.31
Warning			
13.	Do not smoke	USP	88.46
Pregnancy & Breastfeeding			
33.	Do not take if pregnant	USP	94.23
Storage			
42.	Do not store medicine where children can get it	USP	92.31
89.	Do not freeze	USP	90.38
Instruction before/after administer			
56.	Wash hands	USP	100
103. 1	Drink additional water	USP	86.54
Etc			
14.	Check your pulse	USP	96.15
65.	Get emergency help	USP	96.15
Indication			
52.	For headache	FIP	98.08
64.	For headache	USP	90.38
11.	For headache	USP	88.46

### Pictograms that require improvement

The researchers also collected the pictograms that failed but were in the upper quartile (top 25% based on correct score), as these pictograms showed potential for use but might need improvement to increase understanding. Pictograms are compiled in Table 2.

**Table 2:** Pictograms that Failed but have an upper quartile score

Pictogram ID # and Image	Meaning	Source	%Patients correctly answer	Average time (seconds)
Administration				
18.	Wash hands/Place drops in lower eyelid/Wash hands again	USP	84.62	4.10
19.	Wash hands/Place drops in ear/Wash hands again	USP	80.77	4.81
23.	Place drops in ear	USP	84.62	5.38
Regimen				
28.	Take 2 h after meals	USP	80.77	3.65
73.	Take 3 times a day	USP	84.62	3.63
84.	Take with milk	USP	78.85	4.42
95.	Take in the morning	USP	78.85	4.17
Warning				
34.	Do not drink alcohol while taking this medicine	FIP	80.77	3.83
46.	Poison	USP	82.69	3.58
Pregnancy & breastfeeding				
57.	Do not take if breastfeeding	USP	80.77	3.40
92.	Do not take if breastfeeding	FIP	82.69	3.73

#### Pictograms that require precautions

The pictograms that must be avoided or used with caution were the pictograms that failed and were in the lower quartile. (These pictograms are shown in Additional file 1: S1.) Notably, pictograms providing instruction for female organs (#32, #69, and #75) or private areas (#81, #85, #99, and #12) landed in the bottom quartile. Another set of pictograms that were not well understood by Filipino participants was instructions related to chewing, dizziness, take until gone, and do not drink alcohol while taking medicine. (Please see pictograms in Additional file 1: S2.)

Extra precaution must be exercised in pictograms related to pregnancy and children as the comprehension scores were at the bottom quartile. These pictograms (#36, #60, #70, and #74) were targeted to a vulnerable population; thus, this could lead to higher detrimental effects to patients if the pictograms were misinterpreted. (Please see pictograms in Additional file 1: S3.)

Pictograms that were prone to misinterpretation by patients were the pictograms that the participants responded to within 5 s (1/4 of the allotted time) but gave wrong answers. The participants seemed confident with their responses but wrongly interpreted the information which could be dangerous. The most common wrong responses are summarized in Table 3.

**Table 3:** Pictograms that were misinterpreted by patients

Pictogram ID # and Image	Meaning	Source	%Patients Correctly Answer	Average time (seconds)	Most common wrong responses
Administration					
98.	For pulmonary problem	FIP	34.62	4.54	Inhale medicine and Nebulizer
32.	Wash hands/Insert into vagina/Wash hands again	USP	34.62	4.76	Wash hands before and after. Not sure about the center picture
69.	Insert into vagina	USP	17.31	4.54	Insert Intra uterine device (IUD)/Tampon/ Suppository
85.	Insert into rectum	USP	42.31	4.60	Insert suppository
Regimen					
3	Take 2 times a day with meals	USP	26.92	4.94	Take medicine after eating
Pregnancy & breastfeeding					
60.	Are you breastfeeding?	USP	9.62	3.77	Breastfeeding
70.	Are you pregnant or do you plan to become pregnant?	USP	1.92	3.33	Pregnant
Side effect					
9. 5	This medicine may make you dizzy	FIP	42.31	3.37	Headache
104.	This medicine may make you dizzy	FIP	34.62	3.79	For Insomnia, For Sleepy

### Factors associated with pictogram comprehension

The median score of Filipinos was 71 out of 108 pictograms with a median time per pictogram of 3.78 s. The score ranged from 10 to 96, while the median time per pictogram ranged from 2.06 to 14.05 s. Participants were divided into three subgroups based on gender, educational level, and age. In terms of gender, there were 57.69% Female (*n* = 30) and 42.31% male (*n* = 22). The analysis found that there was no statistically significant difference between the total score of the two groups. However, female participants took a statistically significant longer rank-sum average time when viewing the pictograms before giving the response 910 s compared to 468 s for males as shown in Table 4.

**Table 4:** Subgroup analyses of factors associated with pictogram comprehension

	Rank-sum of total score	*Z* value	*p* value*	Rank-sum of average time	*Z* value	*p* value*
Based on gender						
Male (*n* = 22)	661.5 (583)	1.455	0.146	468 (583)	− 2.130	0.033
Female (*n* = 30)	716.5 (795)			910 (795)		
Based on education level						
> Grade 12 (*n* = 26)	952.5 (689)	4.825	< 0.001	600.5 (689)	− 1.620	0.105
≤ Grade 12 (*n* = 26)	425.5 (689)			777.5 (689)		
Based on age						
18–45 Yr (*n* = 26)	763.5 (689)	1.364	0.173	479.5 (689)	− 3.834	< 0.001
≥ 46 Yr (*n* = 26)	614.5 (689)			898.5 (689)		

*Wilcoxon Rank-sum Test

In terms of education level which there were equal samples per group, the rank-sum of the total score was statistically significantly different. The participants below Grade 12 had a lower score as shown in Table 4. The rank-sum for patients with higher education levels was 952.5 compared to only 425.5 for below Grade 12 education when both had an expected value of 689 (*p*-value = 0.0000). For age, there was no statistically significant difference between the total score of the two groups; however, older participants took a statistically significant longer rank-sum average time when viewing the pictograms before giving the response 898.5 s compared to 479.5 s when both have an expected value of 689, as shown in Table 4.

A univariate analysis was performed to confirm the factors that were significantly associated with the participant’s total score shown in Table 5. The result found that the two factors that were significantly associated were education with a Beta value of -25.31(SE = 4.52, *p*-value < 0.0001) and a constant of 77.57 and average time in seconds also significantly associated with the Beta value of -4.89 (SE = 1.01, *p*-value < 0.0001) with a constant of 86.86. This indicated that participants who had an education of less than Grade 12 and those who took longer to give a response had a lower score. With the non-probability samples of 52 participants, the variables that had an impact on comprehension score when all factors were combined in the same equation are summarized in Table 5. The effect seems to be lessened in gender (from -8.8 to -5.8) and age (from − 8.92 to − 1.2) when average time in seconds was included in the multivariate analysis.

**Table 5:** Simple and multiple linear regression assessing factors related to the total score

	Univariate				Multivariate		
	Coefficient	SE	*p*-value	Univariate constant	Coefficient	SE	*p*-value
Gender (Male = reference)	− 8.8	5.69	0.129	70	− 5.85	4.24	0.174
Education (> Grade 12 = reference)	− 25.31	4.52	< 0.001	77.57	− 21.58	4.22	< 0.001
Age (< 45 yr = reference)	− 8.92	5.62	0.119	69.38	− 1.20	4.52	0.793
Average time in seconds	− 4.89	1.01	< 0.001	86.86	− 3.06	1.05	0.006
Multivariate Constant					93.41	4.55	< 0.001

The multivariate model suggested that male participants, in a younger age and higher education, were expected to have the highest average total score of 93.41. The model predicted that the score was expected to decrease by 5.85 points if the user was female, 21.58 points if below Grade 12 education, and 1.20 points if greater than 46 years old. The score also decreased by 3.06 points per every increase in 1 s in the average time before responding. Education level and average time were identified as the significant predictors. This result was consistent with the result of the Wilcoxon rank-sum test performed, where those who have below Grade 12 education had the lower score and it took them longer average time before responding.

## Discussion

Based on the results of the study, there were only 17 pictograms that were most likely to be effective when used in Filipino adults as a stand-alone material. This result proved that even in internationally validated pictograms, added care was still required when used as a sole communication resource. As other researchers suggested, it was important to not disregard other forms of patient communication and information and should consider pictograms as a complement to other forms of patient counseling [4, 8].

Common characteristics of the pictograms that garnered the highest score were the ones that represented a simple message with a pictogram that is commonly encountered by Filipinos. For example, the warning pictogram with the highest score “do not smoke” (88.46%) was commonly used in public places because of Executive Order No. 26, entitled Providing for the Establishment of Smoke-Free Environments in Public and Enclosed Places [21]. For pictograms for pregnancy and breastfeeding, “do not take if pregnant” (94.32%) was an image of a pregnant woman that was similar to what was used in common health education textbooks in primary school. For instruction before/ after administration, the highest scored pictogram “Wash hands” (100%) was commonly seen in public places, such as schools, restrooms, and hospitals. The pictogram “get emergency help” (96.15%) was a common symbol for hospital emergency rooms and emergency response in the Philippines. This was consistent with the findings of Liu, Hoelscher, and Gruchmann (22) that the application experience of the users of the pictogram significantly influenced the target users’ comprehension.

A characteristic of pictograms that required improvement (below 85% but in the upper quartile) was that their messages were more complex in that they required a combination of two or more pictograms. For example, the pictogram for washing of hands was 100%, and the drop in the lower eyelid was 86.54% (both passed), but the comprehension score of pictogram #18, comprising a combination of pictograms, was lowered to 84.62% (failed). This was also true with pictograms #19 and #28 which bundled two or more pictograms together. For complex pictograms, it was still best to complement these pictograms with both verbal and written instructions.

In the case of pictogram #46 “Poison,” some patients identified it as “death,” which was a related term but not exactly correct. For the others, participants were not able to identify key details that gave context to the pictogram like “do not” in pictograms #34, #57, and #92. We could state, therefore, that while some participants had a general idea about what the pictogram was trying to communicate, their responses were either too specific or too general. Overall, they failed to convey the real meaning of the pictogram. The researchers recommended that patient counseling and/or health education should be given to patients to complement the use of pictograms.

Pictograms related to reproductive organs and rectum all landed in the bottom quartile. One possible explanation was that Filipinos are majority Christians and in the country where it was culturally taboo to discuss sexual and reproductive topics resulting in the unfamiliarity of the imagery and modality shown in the pictograms [23]. By extension, extra precaution must be exercised in pictograms related to pregnancy and children because of the potential higher detrimental effects to patients if they were misinterpreted.

The median score of Filipinos of 71 pictograms was comparable to the result of the study conducted in Portugal but the variability of the score was wider in the Philippines, the lowest score was 10, and the highest score was 96 out of the perfect score of 108 [4]. This result suggested that factors contributing to the variability must be investigated. According to the recommendations of the International Pharmaceutical Federation (11), it was important to identify those who may be at risk (lower comprehension) as this would allow the health care providers to apply specific communication techniques that best suit the needs of the patients.

Time taken to comprehend a pictogram should be as low as possible as a pictogram was developed to convey quick and clear information without language or words. The median average time taken to comprehend pictograms of Filipinos was 3.78 s (IQR 2.06–14.05 s), which showed that pictograms were quickly comprehended by the participants. However, most responses were incorrect. This indicated the importance of incorporating written text into the pictograms and the need for further development of local pictograms to ensure better comprehension.

In terms of the subgroup analyses conducted, for education level, the result suggested that higher education translated to a higher comprehension of pictograms. In the Philippines, since the USP and FIP pictograms were not commonly used and were not included in the high school curriculum, the comprehension score was expected to be low. To increase comprehension, previous studies suggested that training and patient education were necessary to ensure the effectiveness of symbols [2, 22, 24]. The researchers, therefore, recommended the inclusion of topics related to health pictograms in basic health education in the Philippines. Alternatively, another strategy from the literature suggested that verbal instructions were better comprehended by individuals with low literacy skills than pictograms. Therefore, practitioners must not use the pictograms alone but must be complemented with verbal and written instructions during patient counseling [8].

For gender, there was no statistically significant difference between males and females, but it was noted that female participants tended to have a longer average time before giving a response. This was consistent with previous studies in the Philippines that demonstrated high priority on the promotion of gender equality [25]. Filipino women may be considered as one of the most advanced vis-à-vis the women in other countries, in the areas of academic, professional, politics, and legislation [25].

For age, interestingly, there was also no difference in terms of score but only on the average time before giving a response. This was not consistent with the study conducted previously [15]. One possible reason was that elderly patients tended to have a lower score in other studies, because of possible visual impairment [26]. This was not a factor in the current research because the pictograms were printed largely on the flipbook during data collection. In line with this, the researchers have two recommendations. First, to effectively use the pictograms, the larger ones are preferred to account for the patient’s visual acuity [26]. Second, there is a need to re-test the pictograms in their intended actual size in practice to ensure effectiveness.

It should be noted, however, that the limitation of this study was the sample size of 52 participants which was computed specifically for the major objective. This may not be enough to definitively assess the subgroup analyses for gender and age. With the current sample size, the retrospectively calculated power was only 32.65% for gender and 34.22% for age. For future researchers who wish to assess these factors with a power of at least 80%, the recommended sample size is *N* = 168 (*N* per group 84). Other factors might affect comprehension scores that were not explored in this research, such as occupation, income, access to health information materials, access to the internet, and participation in health programs among others.

The other limitations of this study were that the participants were only limited to the national capital region of the Philippines. Data collected from the national capital region were a good representative of the whole country since it is a melting pot of different regions; however, it is still recommended to conduct a similar comprehension test per region. The Philippines, being an archipelago of 7100 islands, has different cultures, languages, practices, and socioeconomic statuses in each region. The data collection tool must also be improved to reflect the intended size of the pictogram and the intended dialect when used in patient information material.

In summary, the researchers through the findings offered the following strategies and recommendations for future researchers to improve the comprehension of pictograms in the Philippines:

1. In a set of pictograms for the same purpose, select the pictograms with the highest comprehension score for utilization in PIL regardless of the source.
2. Complement the pictograms with written instructions and verbal reinforcements to ensure comprehension especially when dealing with patients below Grade 12 [19, 27].
3. Develop illustrations adapted to the Filipino culture with designs that follow the best practice principles in written health education material design and standards [20, 28].
4. Retest the pictograms at their intended print size with texts in the intended dialect to ensure comprehension before mass utilization on the target population. [27]
5. Retest pictograms in a larger sample size *N* = 168 (84 per group) and randomly sampled from different regions of the Philippines for better generalizability of result.

In terms of public health intervention, pictograms should be included in the Philippines’ basic health education (Grade 11 or Grade 12) to effectively and safely use them in patient information materials. Much effort is needed to educate the public with reproductive health-related pictograms.

## Conclusions

In conclusion, only 17 pictograms out of 108 (15.74%) were recommended to be used as a stand-alone material. The median score of Filipinos was 71 out of 108 pictograms (IQR = 10–96). The education level of the patient was the most notable factor associated with the comprehension of pictograms in Filipino patients, where the score is significantly lower if the participant was below Grade 12 in education level. Therefore, the researchers recommended the use of verbal and written instructions to complement pictograms to enhance comprehension. Furthermore, the government should consider the inclusion of health pictograms in basic health education.

## Supplementary Information

**Additional file 1:** **S1.** Pictograms that Failed and in the lower quartile score. **S2. **Notable Pictograms that Failed and in the lower quartile score. **S3. **Pictograms for special population that Failed and in the lower quartile score.

## Data Availability

The datasets used and/or analyzed during the current study are available from the corresponding author on reasonable request.

## Abbreviations

USP: United States Pharmacopeia
FIP: International Pharmacy Federation
ANSI: American National Standards Institute
SD: Standard deviation
FDA: Food and drug administration
PIL: Patient information leaflet
NCE: New chemical entities
PI: Package insert
GHS: Globally Harmonized System
UPMREB: University of the Philippines Manila Research Ethics Board
