# Supplementary material for: Evaluation of pharmaceutical pictogram comprehension among adults in the Philippines
Source: J Pharm Policy Pract. 2022 Apr 7;15:30. doi: 10.1186/s40545-022-00426-y (PMC8991701; doi:10.1186/s40545-022-00426-y)
Supplement: Supplementary file 1 — Additional file 1: S1. Pictograms that Failed and in the lower quartile score. S2. Notable Pictograms that Failed and in the lower quartile score. S3. Pictograms for special population that Failed and in the lower quartile score. [file 40545_2022_426_MOESM1_ESM.docx]

**Additional file 1**

**S1. Pictograms that Failed and in the lower quartile score**

| Pictogram ID # and Image | Meaning | Category | Source | %Patients Correctly Answer | Average  Time (seconds) |
| --- | --- | --- | --- | --- | --- |
| 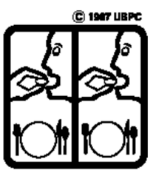3. | Take 2 times a day with meals | Regimen | USP | 26.92 | 4.94 |
| 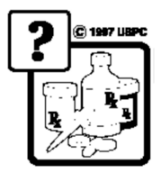6. | Are you taking any other medicine? | Etc. | USP | 0.00 | 6.04 |
| 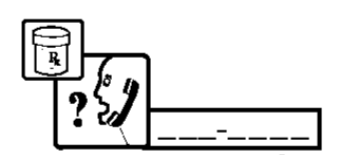22. | If you have questions, call this number. | Etc. | USP | 42.31 | 5.38 |
| 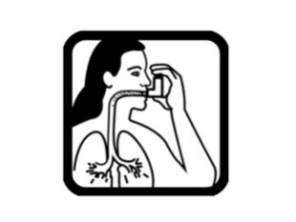98. | For pulmonary problem | Administration | FIP | 34.62 | 4.54 |
| 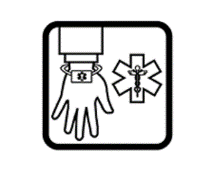108. | Wear medical alert | Etc. | USP | 13.46 | 6.65 |
| 35. | Do not store near heat or in sunlight | Storage | USP | 30.77 | 5.27 |
| 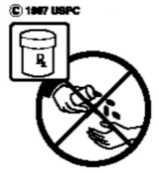30. | Do not share your medicine with others | Warning | USP | 7.69 | 6.04 |
| 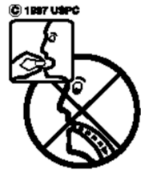43. | Do not swallow | Warning | USP | 38.46 | 6.12 |
| 45. | Do not take other medicines with this medicine | Warning | USP | 15.38 | 5.98 |
| 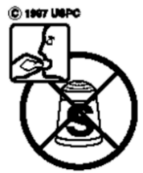79. | Do not use additional salt | Warning | USP | 36.54 | 5.27 |

S2. Notable Pictograms that Failed and in the lower quartile score

| Pictogram ID #  and Image | Meaning | Category | Source | %Patients  Correctly Answer | Average  Time (seconds) |
| --- | --- | --- | --- | --- | --- |
| 32. | Wash hands/  Insert into vagina/Wash hands again | Administration | USP | 34.62 | 4.76 |
| 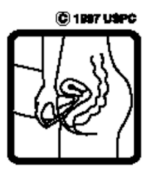69. | Insert into vagina | Administration | USP | 17.31 | 4.54 |
| 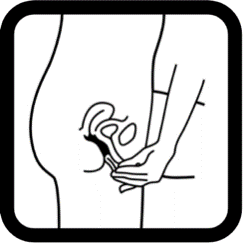75. | Insert into vagina | Administration | FIP | 17.31 | 5.46 |
| 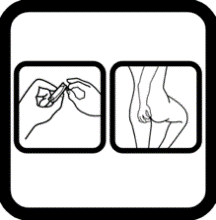81. | Insert into rectum | Administration | FIP | 36.54 | 5.10 |
| 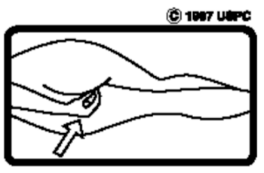85. | Insert into rectum | Administration | USP | 42.31 | 4.60 |
| 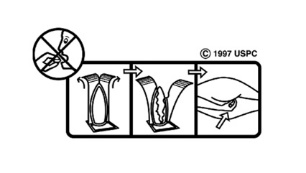99. | Remove foil from suppository before inserting into rectum | Administration | USP | 26.92 | 7.23 |
| 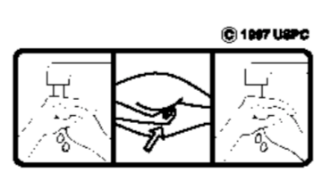12. | Wash hands/Insert into rectum/Wash hands again | Administration | USP | 42.31 | 6.54 |
| 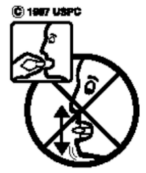67. | Do not chew | Warning | USP | 38.46 | 5.90 |
| 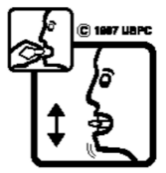72. | Chew | Instruction before/after administer | USP | 15.38 | 5.25 |
| 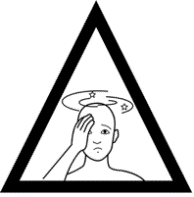59. | This medicine may make you dizzy | Side effect | FIP | 42.31 | 3.37 |
| 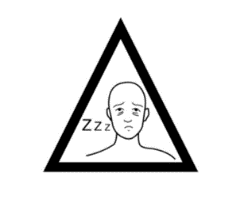104. | This medicine may make you dizzy | Side effect | FIP | 34.62 | 3.79 |
| 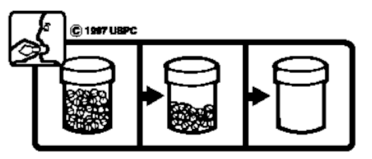37. | Take until gone | Etc. | USP | 36.54 | 5.44 |
| 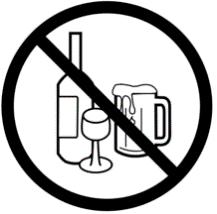34. | Do not drink alcohol while taking this medicine | Warning | FIP | 80.77 | 3.83 |

S3. Pictograms for special population that Failed and in the lower quartile score

| Pictogram ID #  and Image | Meaning | Category | Source | %Patients Correctly Answer | Average  Time (seconds) |
| --- | --- | --- | --- | --- | --- |
| 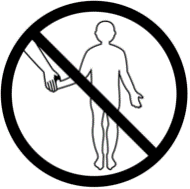36. | Do not use this medicine in children | Warning | FIP | 13.46 | 5.96 |
| 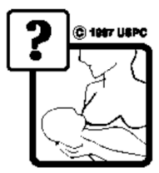60. | Are you breast-feeding? | Pregnancy & Breastfeeding | USP | 9.62 | 3.77 |
| 70. | Are you pregnant or do you plan to become pregnant? | Pregnancy & Breastfeeding | USP | 1.92 | 3.33 |
| 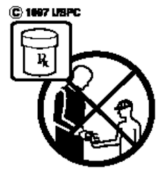74. | Do not give medicine to children | Warning | USP | 28.85 | 5.13 |
